# Recent Developments in Minimally Invasive Cardiac Surgery: Evolution or Revolution?

**DOI:** 10.1155/2015/483025

**Published:** 2015-10-08

**Authors:** Antonino G. M. Marullo, Francesco G. Irace, Piergiusto Vitulli, Mariangela Peruzzi, David Rose, Riccardo D'Ascoli, Alessandra Iaccarino, Angelo Pisani, Carlotta De Carlo, Giuseppe Mazzesi, Antonio Barretta, Ernesto Greco

**Affiliations:** ^1^Department of Medico-Surgical Sciences and Biotechnologies, Sapienza University of Rome, Corso della Repubblica 79, 04100 Latina, Italy; ^2^Department of Cardiovascular, Respiratory, Nephrological, Anesthesiological, and Geriatric Sciences, Policlinico Umberto I, Sapienza University of Rome, Viale del Policlinico 155, 00161 Rome, Italy; ^3^Cardiothoracic Department, Lancashire Cardiac Centre, Blackpool Victoria Teaching Hospital, 38 Whinney Heys Road, Blackpool FY3 8NR, UK; ^4^Department of Cardiac Surgery, Ospedale dell'Angelo, Mestre, Via Paccagnella 11, 30174 Venice, Italy

## Abstract

Intraluminal aortic clamping has been achieved until now by means of a sophisticated device consisting of a three-lumen catheter named Endoclamp, which allows at the same time occlusion of the aorta, antegrade delivering of cardioplegia, and venting through the aortic root. This tool has shown important advantages allowing aortic occlusion and perfusate delivering without a direct contact with ascending aorta reducing meanwhile the risk of traumatic and/or iatrogenic injuries. Recently, a new device (Intraclude catheter) with the same characteristics and properties has been proposed and introduced in clinical practice. The aim of this paper is to investigate the differences between Endoclamp and Intraclude catheters and to analyze the advantages advocated by this new device for intraluminal aortic occlusion since it is noticeable as these new technological tools are gaining more and more attractiveness due to their appraised clinical efficacy.

## 1. Introduction

Since Bailey reported in 1951 the first surgical treatment of mitral valve with mitral annulus narrowing by external constriction through left thoracotomy [1], several approaches and techniques for mitral valve surgery have been progressively proposed, modified, and refined, especially after the introduction of CPB (cardiopulmonary bypass). LILLEHEI and colleagues reported the first case of mitral valve repair through a right thoracotomy, using femoral artery cannulation for cardiopulmonary bypass (CPB) [2]. With extensive use of CPB in 1960s, median sternotomy became the primary surgical approach to mitral valve considering the undoubted advantages related to its reliability, speed, and worthy exposure of the mitral valve as well as access to the rest of the heart compared to right thoracotomic incision. In the late 90s, the increasing interest for minimally invasive surgery due to patients demand, marketing policy, and new developing technologies stimulated the reconsideration of different left atrial and mitral approaches. At first, parasternal incision and partial sternotomy, following the work of Gillinov and Cosgrove [3], have been the most popular minimally invasive approach. More recently, in accordance with the rule of courses and historical claims, the experimental works performed in the laboratories at Stanford University and New York University have refocused the attention to right thoracotomy leading to the development of minithoracotomic video-assisted or video-guided port-access approach [4].

This new technique exploited a peripheral perfusion and a balloon catheter for aortic occlusion allowing a less invasive procedure through a mini thoracotomy (4–6 cm) approach [5–7], with undoubted advantages related to an overall reduction in surgical trauma, an effective improvement in patient comfort, lower morbidities, and shorter in-hospital stay, besides the remarkable cosmetic advantages especially in women [8–10].

Due to materials and instrumentation improvements different cannulation and aortic clamping strategies are nowadays available:

- Full extra-thoracic CPB with external transthoracic aortic clamping.
- Full extra-thoracic CPB with endoaortic clamping.
- Central arterial cannulation with external transthoracic aortic clamping.
- Central arterial cannulation with endoaortic clamping.

Intraluminal aortic clamping has been achieved until now by means of a sophisticated device consisting of a three-lumen catheter named Endoclamp, which allows at the same time occlusion of the aorta, antegrade delivering of cardioplegia, and venting through the aortic root. This tool has shown important advantages allowing aortic occlusion and perfusate delivering without a direct contact with ascending aorta reducing meanwhile the risk of traumatic and/or iatrogenic injuries. Therefore, other than in less invasive surgery procedures, the Endoclamp can be successfully adopted in systematic surgery especially in presence of extensive calcification of ascending aorta and in redo procedures allowing safer cross-clamping without requirement for dissection and manipulation of the ascending aorta and aortic root. Recently, a new device (Intraclude catheter) with the same characteristics and properties has been proposed and introduced in clinical practice. The aim of this paper is to analyze the differences between Endoclamp and Intraclude catheters and to analyze the advantages advocated by this new device for intraluminal aortic occlusion since it is noticeable as these new technological tools are gaining more and more attractiveness due to their appraised clinical efficacy.

## 2. Surgical Technique Using Endoclamp

**Figure 1:**
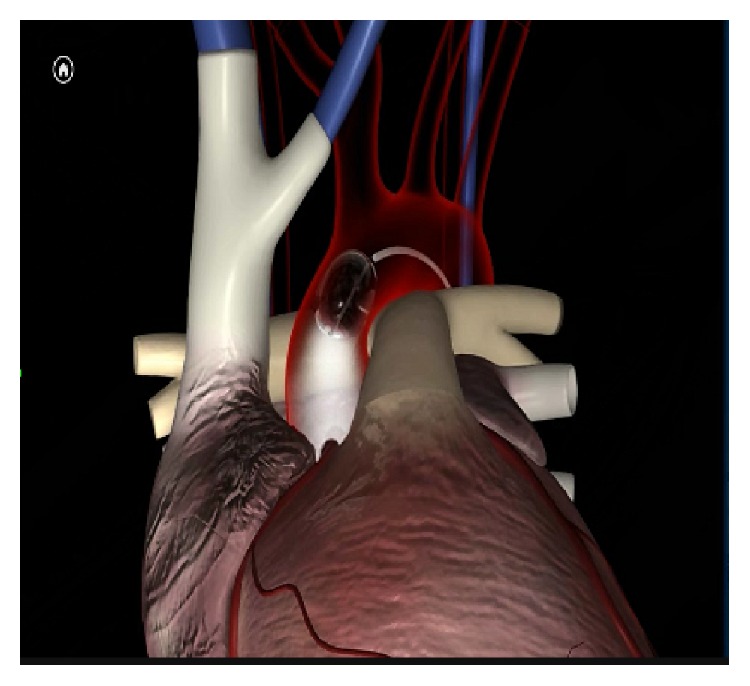
Endoaortic balloon correct placement.

**Figure 2:**
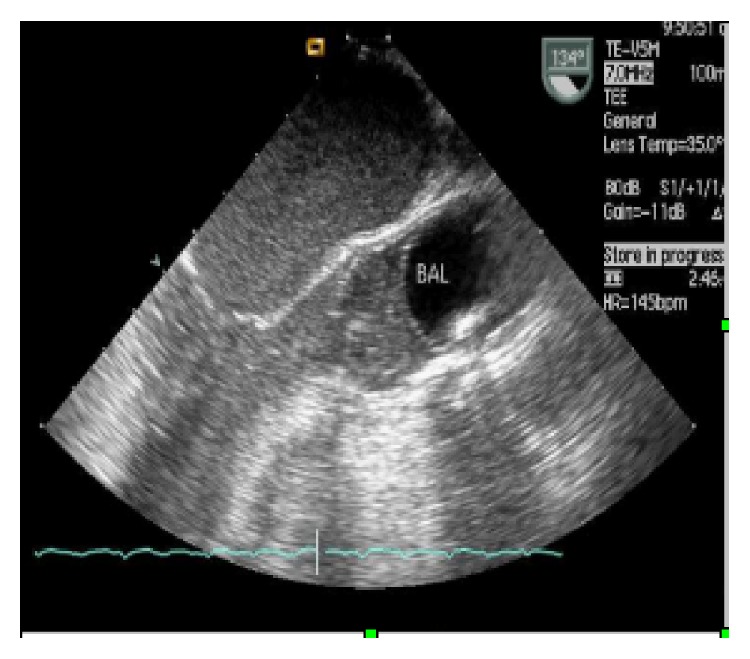
TEE monitoring of balloon position.

Under general anesthesia, the patient is positioned in the supine position, with slight elevation (30°) of the right hemithorax. All candidates for minithoracotomic video-assisted or video-guided port-access approach are ventilated with a double-lumen endotracheal tube in order to exclude, when needed, right lung ventilation. Monitoring includes double side arterial lines and use of TEE (Transesophageal Echocardiography). A small right mini thoracotomy (working port) and two additional ports are performed as previously described [4, 11–13]. Venous drainage is generally achieved with double venous cannulation with a 14–20 Fr cannula placed percutaneously, under transesophageal echocardiographic guidance, through the internal jugular vein into the superior vena cava and a cannula into the inferior vena (Fr) cava through the femoral vein using Seldinger technique. Arterial cannulation is performed with placement under direct vision into the femoral artery of a dedicated 21–23 Fr cannula (Endoreturn), a Y-shaped device with a side branch that allows the introduction of the occlusion balloon. The Endoclamp endoaortic balloon is at this time placed from the Endoreturn cannula side branch, under TEE guidance, in the ascending aorta just above the sinotubular junction (Figures 1 and 2).

The Endoclamp is a 10.5 Fr, 100 cm long, three-lumen catheter with an elastomeric balloon near its tip customized for endoluminal occlusion of the ascending aorta in order to separate the aortic root from arterial circulation. The surface contact of the balloon with the aortic wall is limited to 10 mm in length to avoid coronary occlusion during cardioplegia delivery. The large central lumen of the catheter attends two functions: delivery of cardioplegic solution during occlusion and venting from the left cardiac chambers both through the aortic root. The two remaining lumens are designed to serve as conduits for balloon inflation and aortic root pressure monitoring throughout the cardiac arrest. After proper position of the device, CPB is instituted and, under TEE monitoring to avoid balloon migration, the endoaortic balloon is progressively inflated with careful attention to its position at the level of the sinotubular junction [14]. Once its correct position is ascertained the cardioplegic solution is administered via an antegrade route. Using this technique blood pressure through the arterial line should be continuously monitored in order to promptly detect possible modifications that might be suggestive of partial or transient occlusion of the arterial arch vessels. In our experience the balloon should initially be inflated using an amount of saline solution proportional to the diameter of the sinotubular junction (1 : 1) to avoid unnecessary overexpansion. The balloon pressure is continuously monitored to reach and maintain a target pressure of around 350/400 mmHg. It is important to remember that during the cardiac arrest the Endoclamp pressure can progressively decrease by 10–20% due to the variation of temperature and the reduced stiffness of the aortic wall. In this case, no additional volume of intraballoon saline is needed if the heart is asystolic and the field is dry. The adhesion of the balloon with the aortic wall is crucial for steadiness of the device. Usually a balance is achieved because the balloon is pushed downstream by arterial flow from femoral arterial cannula and upstream by the pressure originated inside the root by cardioplegia delivery or by the systolic ejection from the heart before complete cardiac arrest. In presence of trivial aortic regurgitation and/or inadequate drainage of the left ventricle with unsatisfactory cardiac arrest during the antegrade cardioplegic induction, adenosine injection directly in the aortic root can be used to facilitate heart arrest and therefore facilitate the correct endoclamping function. In addition, to optimize left ventricular drainage, an Endopulmonary Vent Catheter, previously inserted by anesthesiologist through central vein access, can be used when needed. During surgery continuous TEE monitoring is recommended, to provide an optimal monitoring of venous cannula position, deairing maneuvers, and, certainly, an assessment of valve function after the operation [15, 16]. Other indirect monitoring tools include NIRS (INVOS) or transcranial Doppler [17] both able to detect any functional impairment of cerebral blood flow [18] caused by balloon migration. Once the surgical procedure has been completed, the balloon is deflated and partially withdrawn. At this time aortic venting is achieved through the same sideline of the catheter. After weaning from CPB, the device is fully removed through the side branch of the Endoreturn arterial cannula. A major concern of this technique with adoption of this device is the possible reduction of the arterial cannula lumen after introduction of the endoluminal balloon. Although this phenomenon is unusual with a 23 Fr cannula it has been described with the 21 Fr size with possible negative impact on systemic perfusion or in safety of CPB management. The reduction of the arterial cannula lumen related to the steric hindrance of the Endoclamp catheter can, in fact, result in an elevated pressure on the line of arterial perfusion (>250 mmHg), especially in cases of small and elastic femoral arteries, like in young women with small body surface area or in patients with severe atherosclerotic disease of the iliac-femoral tree. In our experience, with pressure >300 mmHg during full flow CPB, a contralateral femoral arterial cannulation, even with a small (18-19 Fr) cannula, is advisable by means of a double Y line perfusion to avoid malperfusion or complication on CPB lines or oxygenator.

## 3. Pitfalls

Although most of studies showed the feasibility and safety of minimally invasive mitral valve surgery using the endoaortic clamping technique [19–21], several specific issues emerged from data reported in literature. Particularly in first series, multiple severe complications have been described, such as aortic dissection or iliac artery injury [22], probably due to first generation stiffer catheters, worse monitoring techniques, and learning curve of the operator. In fact, originally Endoclamp position monitoring was performed using fluoroscopy only during the positioning of the device without any further control during the surgical procedure and, moreover, surgeons were not enough skilled to manage catheters and guide-wires. Nowadays severe vascular complications are very rare and cannot be considered a specific burden of endoluminal clamping technique itself [23, 24].

Concerning generic complications some authors and especially the ISMICS (International Society for Minimally Invasive Cardiothoracic Surgery) summit claimed an augmented risk for cerebrovascular events, hypothetically due to greater use of femoral arterial cannulation for CPB. Plaque embolization during catheter introduction into the femoral artery or related to retrograde perfusion as well as traumatic injuries with consequent artery dissection or pseudoaneurysms formation and epiaortic vessel obstruction caused by balloon migration are well known complications [25–27] limited in more recent practice by the adoption of a more careful monitoring and dedicated catheter and devices.

Furthermore, another pitfall can be related to insertion of the endoluminal balloon into the arterial cannula that in some cases can induce increased resistance in the arterial line requiring a double arterial cannulation [28].

Other possible complications, described particularly in the first “era,” are directly related to the endoluminal balloon device. Retrograde aortic dissection, balloon migration, balloon caught by suture for proximal anastomosis in coronary surgery, and balloon perforation during mitral valve procedure have been described and reported in literature [29]. Other authors, despite the overlapping results between classical sternotomy technique and port-access technique using Endoclamp, described cases that required switching to external cross-clamping to solve the unexpected problems arisen with endoluminal balloon [30].

## 4. Intraclude Improvements

In this milieu a novel device, the Intraclude catheter, has been designed and approved for clinical use to overcome and solve these issues. Intraclude is a three-lumen catheter designed as an evolution with the same purposes of the Endoclamp. Innovations of this device compared to the Endoclamp are related particularly to the catheter size. New developed technology permitted, in fact, reducing the size of the device from 10.5 Fr to 9.5 Fr leading to the attainment of decreased resistances through the arterial cannula and, therefore, allowing a major blood flow at minor pressures, with consequent limitation of the stress and the so called “sand blast effect” related to the high-pressure blood jet. Some authors reported occasionally experiences of catheter “kinking,” probably due to the minor caliber and softer material with respect to Endoclamp. This phenomenon is not frequent since the reduced caliber of the tip of Intraclude compared to the proximal part (hub) that maintains a diameter of 10.5 Fr is specifically designed to avoid the risk of kinking or twisting of the catheter and consequently to prevent the hazard of high pressure during cardioplegia delivering. Femoral artery injury or pseudoaneurysm formation leading to limb ischemia could be complications of femoral cannulation itself, regardless of the endoaortic balloon use, and prevention strategies are described elsewhere [27, 31]. Concerning balloon migration, Intraclude has a different shaped balloon with wider cylindrical-shape compared to the spherical balloon of the Endoclamp with advantages in terms of surface contact that is increased from 10 mm to 18 mm. This change is supposed to ameliorate the stability of the endoluminal balloon with a better “fitting” into the aortic lumen and improved adhesion to the aortic wall allowing a more reliable sealing after inflation and therefore reducing the incidence of dislocation and/or blood leak into the ascending aorta.

To further ameliorate safety and performance of the endoluminal clamping, the Intraclude shaft is curve-shaped, allowing a better adhesion to the aortic arch and a perfect tip orientation towards the aortic valve for the cardioplegia delivery. Moreover, this curvature is supposed to avoid the “slack effect” experienced with the straight Endoclamp shaft limiting the possible migration of the balloon toward the aortic valve due to the tension generated by the catheter bended into the aortic arch.

The different balloon shape also allows the availability of a wider range of calibers, ranging from 20 to 40 mm, rather than the Endoclamp limited to a range of 20–38 mm. Dealing with balloon disruption or perforation, continuous TEE monitoring is essential to avoid malposition and prevent accidental perforation of the balloon during mitral valve surgery as well described in literature [14]. To prevent spontaneous ruptures, the inflation volume has been reduced to 35 mL (from 40 of Endoclamp), preventing in this way either possible clamp failure, or balloon migration. In this regard, it is useful to keep in mind that pressure variations throughout the surgical procedure can lead to migration of the device as well as that migration itself can determine pressure change with vicious cycle mechanism.

In a recent analysis conducted by several European surgeons [32], the routine adoption of Intraclude device has been considered as one of the key factors leading to a significant reduction of morbidity and complications with particular emphasis concerning the stroke rate incidence.

## 5. Discussion

**Figure 3:**
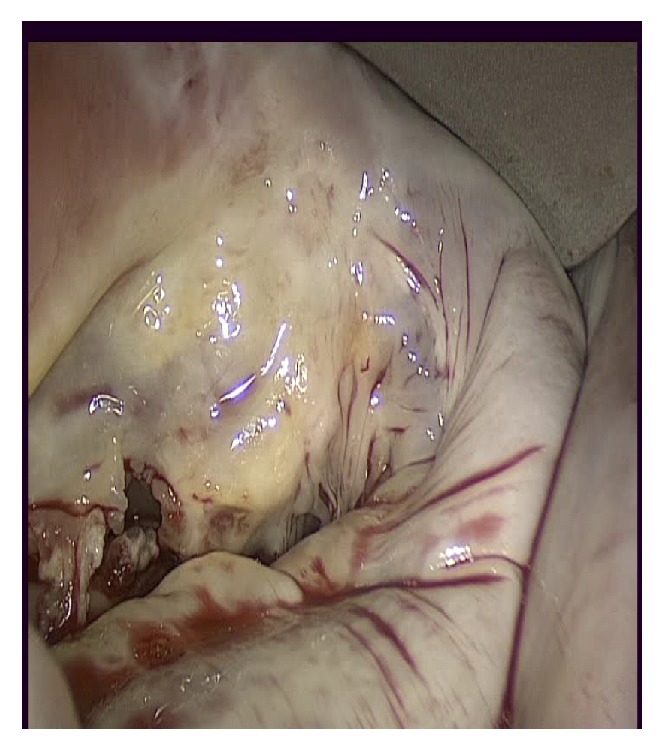
Excellent visualization of the mitral valve by Thru-Port System.

Minimally invasive surgical treatment of valvular heart disease has steadily increased over the last several years becoming an established technique with high successful outcome in many specialized centers. The vast majority of larger clinical studies demonstrate that minimally invasive valve surgery using the port-access approach after an initial learning curve [19, 20, 33–36] is a safe and effective approach in terms of short- and long-term results, mainly for redo operations and even for elderly patients with moderately elevated perioperative risk. Furthermore this technique has shown a low morbidity and mortality achieving functional and echocardiographic outcomes comparable to those obtained with conventional surgery. Measurable patient benefits from case-matched control trials [37–39] include less pain, less blood transfusions, fewer wound infections and pulmonary complications, and faster recovery as well as a better cosmetic result. Moreover recent improvements in Thru-Port systems offer excellent visualization of cardiac structures (Figure 3) through a virtually bloodless, unobstructed operative field without any increase of operative difficulty, procedure, and pump times, thus consenting to adopt successfully the same well established surgical techniques through the smallest incision possible.

In this setting the new device Intraclude undoubtedly improved safety and properness of intraluminal aortic occlusion during minimally invasive mitral surgery. The preshaped curved silhouette, the reduced diameter, and the cylindrical balloon profile have shown unquestionable advances allowing an easier and more reliable endoartic clamping with positive impact in terms of reduced stroke incidence suggesting a spreader use of this device other than in minimally invasive surgery. New catheter Intraclude has been introduced in the European market in 2012, replacing the old catheter Endoclamp. Since then, more than 2500 catheters have been used in Europe for ascending aorta occlusion and cardioplegia delivering during minimally invasive mitral valve surgery.

In our experience, we used the catheter Endoclamp from 2000 in our patients in more than 600 operations. We moved to use the new device, Intraclude, from the beginning, in our patients and we performed more than 60 cases with this device. At the beginning of the experience, we had some concerns regarding the extreme softness of the catheter with some risk of twisting and kinking. Nevertheless, we immediately appreciated, with respect to Endoclamp, the fact that its reduced size allowed a minor increase in the arterial line pressure during perfusion. Using Endoclamp we experienced at least five cases of migration of the balloon toward aortic valve and left ventricle. We did not have similar cases with Intraclude probably due to wider adhesion of this balloon to the aortic wall. The preshaped curvature of Intraclude makes its position easier in contact with the aortic arch reducing the need of additional maneuvers to avoid slack of catheter in thoracic aorta. From our experience, during introduction of the device it is mandatory to avoid any twisting of the curvature before the tip of the catheter reaches the root of ascending aorta. We had four cases of spontaneous rupture of Endoclamp but none using Intraclude. This could be related to the different shape of the balloon and/or to the material of this new device that seems to be helpful in terms of strength and fitting of the balloon with irregularities of the aortic wall. In conclusion we firmly believe all these technological developments and tools applied to minimally invasive procedure have gained, over time, more and more attractiveness due to their appraised clinical efficacy, and the acquired clinical experience in thousands of patients worldwide has led to a global improvement and to an implementation of this promising and ground-breaking surgical approach. In brief we are facing a gradual process where something changes into a different and usually more complex or better form, which simply means evolution.
